# Socioeconomic Determinants of Smoking in the Kingdom of Saudi Arabia

**DOI:** 10.3390/ijerph18115665

**Published:** 2021-05-25

**Authors:** Ameerah M.N. Qattan, Micheal Kofi Boachie, Mustapha Immurana, Mohammed Khaled Al-Hanawi

**Affiliations:** 1 Department of Health Services and Hospital Administration, Faculty of Economics and Administration, King Abdulaziz University, Jeddah 80200, Saudi Arabia; aqattan@kau.edu.sa; 2 Health Economics Research Group, King Abdulaziz University, Jeddah 80200, Saudi Arabia; 3 Department of Health Policy Planning and Management, School of Public Health, University of Health and Allied Sciences, Private Mail Bag 31, Ho, Ghana; mkboachie@gmail.com; 4 SAMRC Centre for Health Economics and Decision Science, School of Public Health, University of the Witwatersrand, Johannesburg 2193, South Africa; 5 Institute of Health Research, University of Health and Allied Sciences, Private Mail Bag 31, Ho, Ghana; mustaphaimmurana@gmail.com

**Keywords:** cigarettes, income, intensity, logit, negative binomial, smoking, tobacco

## Abstract

Tobacco consumption, or smoking, is a major cause of preventable morbidity and mortality worldwide, including in the Kingdom of Saudi Arabia (KSA). In order to reduce the consumption of tobacco products, it is necessary to understand the factors that drive it. However, little is known about the socioeconomic determinants of tobacco smoking in the KSA. This study, therefore, investigates the socioeconomic factors that influence tobacco smoking in the KSA. Using a national health survey, the study employs logistic and negative binomial regressions to examine the socioeconomic factors associated with smoking. Moreover, the inequality concentration indices (CIs) are used to assess inequalities in smoking. The results reveal that the smoking prevalence is 14.09% of the 8813 respondents considered in this study. The prevalence of smoking is higher among men (25.34%) than among women (1.91%). On the determinants of smoking, the logistic regression results show that higher income is associated with lower likelihood of smoking. Other factors significantly associated with current smoking status are marital status, gender, age, and region of residence. Similarly, gender, age and region of residence are significantly associated with smoking intensity. As regards the inequality analysis, at the national level, the results show that smoking is concentrated among those with higher socioeconomic status (income: CI = 0.071, *p* < 0.01; education: CI = 0.095, *p* < 0.01), but with significant regional variations. By gender disaggregation, the results also show that the income-based CIs are positive for both males and females, but statistically insignificant. Education-based CI is positive for males and significant (CI = 0.057, *p* < 0.05), while it is negative and insignificant for females during the study period. The findings call for targeted tobacco control measures focusing on those with higher socioeconomic status, as well as factors such as age, gender, marital status and region of residence.

## 1. Introduction

Tobacco kills half of its long-term users. Each year, more than eight million people die from tobacco use (both direct and indirect). Although the harmful health effects of tobacco use are known, many people still consume tobacco products. Thus, globally, there are about 1.3 billion people who use tobacco [1].

In the Kingdom of Saudi Arabia (KSA), 12.2% of the adult population were current smokers and the majority of those smokers (74.1%) smoked 15 cigarettes daily, as of 2013 [2]. Earlier surveys had estimated smoking prevalence to be about 12% [2,3], suggesting that the prevalence of current smoking had not significantly changed for some time. In 2018, the Saudi Food and Drug Authority conducted a survey to update tobacco use information. They found that 21.4% of the adult population smoked, in comparison to 12.2% in 2013, indicating an increase in prevalence between 2013 and 2018 [4]. This indeed remains a major concern because, globally, about 1.2 million deaths occur due to non-smokers’ exposure to second-hand smoke [1]. As such, if the prevalence of current smoking in the KSA has increased from 12.2% in 2013 to 21.4% in 2018, the implication is that the number of non-smokers likely to be exposed to second-hand smoke has also increased significantly.

Although tobacco use provides utility to its consumers, it causes many chronic and non-communicable diseases, such as cancers, chronic obstructive pulmonary disease (COPD), and cardiovascular diseases. In 2018, 10,518 people died of cancer and 24,485 new cases were detected in the KSA [5]. It is estimated that about 70,000 people die annually in the KSA due to smoking-related diseases [6]. Tobacco use imposes a huge economic cost on societies. At the global level, about 1.8% of gross domestic product (GDP) is lost to tobacco use [7]. In the KSA, the cost of tobacco use was approximately USD 20.5 billion between 2001 and 2010 [8] and, in 2012, 0.2% of GDP was lost due to smoking [7]. These costs, emanating from morbidity and mortality, increased to 0.98% of GDP in 2016 [9]. This situation in Saudi Arabia is similar to that in South Africa, where the economic cost of smoking amounted to 0.97% of GDP in 2016 [10]. The above, therefore, makes tobacco use a major global health concern [7].

Due to tobacco’s harmful effects and the cost it imposes on countries, the WHO is assisting governments in developing strategies to combat tobacco use through the WHO Framework Convention on Tobacco Control (FCTC). Although the KSA introduced a national tobacco control program in 2002, the country only intensified its efforts after ratifying the WHO FCTC in 2005 [2,11]. As such, currently, smoking in public places and sales to minors are prohibited [2]. Additionally, for the first time, the country implemented the WHO FCTC Article 6 by introducing an excise tax on tobacco products in 2017, which was revised in 2019 [12]. The KSA is also the pioneer of the “sin tax” in the Gulf region [13].

The effectiveness of the above strategies relies on knowing the factors that influence smoking behavior. A few studies have examined the prevalence and determinants of smoking in the KSA [2,3,14,15]. However, while using nationally representative data enhances the findings’ generalizability, only one study used a nationally representative survey to study smoking patterns, but it did not account for income (or its proxy) [2]. However, income is an important socioeconomic factor influencing smoking decisions [16]. Moreover, some studies estimate that smoking is disproportionately concentrated among people with lower socioeconomic status (SES) (based on income and/or education), although variations exist between countries and gender [17,18,19,20,21]. However, in the KSA, the extent of inequalities in smoking is unknown.

In order to address the above gaps in the literature, we hypothesize that socioeconomic status has statistically significant association with smoking behavior (and its associated inequalities) in the KSA. To test our hypothesis, we use a nationally representative dataset, logistic and negative binomial regressions, as well as inequality concentration indices (Cis). These help in arriving at findings that are more representative of the entire country, as well as revealing whether there are income-level differentials with regard to smoking in the KSA, which would help in designing effective targeted smoking control measures.

The rest of this paper is structured as follows. The methods employed by the study are presented in the next section while, in the third section, the study’s results are shown. The penultimate section discusses these results, while the study’s conclusion is presented in the final section.

## 2. Materials and Methods

### 2.1. Data

The study uses the latest data from the Saudi Health Interview Survey (SHIS), conducted in 2013 by the Saudi Ministry of Health, the Institute for Health Metrics and Evaluation (IHME), and the University of Washington. The survey, which was administered through face-to-face interviews, collected information on health and demographic characteristics in order to assess the prevalence of several chronic conditions and to identify their risk factors, as well as track the health of the population of Saudi Arabia. The survey’s respondents were selected through a multi-stage probability sampling strategy, making it nationally representative. A total of 10,735 individuals aged over 15 years were interviewed out of 12,000 originally contacted households, indicating a response rate of about 90% in the SHIS [22]. A comprehensive description of the sampling method and data can be found elsewhere [23,24]. The dataset has been used to study medication use for chronic health conditions [23], the consumption of foods and beverages [24], tobacco consumption pattern [2], socioeconomic inequalities in diabetes prevalence in the KSA [25], and socioeconomic inequalities in uptake of breast cancer screening among Saudi women [26]. Due to missing data as a result of nonresponses, this study limits the analysis to respondents who have complete information on all the variables of interest. Therefore, this study’s analysis is based on a sample of 8813 respondents.

### 2.2. Variables

The SHIS collected information on cigarette smoking status and the daily number of cigarettes smoked by respondents. In the survey, respondents were asked whether or not they currently smoked any tobacco products, such as cigarettes. This takes the value of one for respondents who smoke (yes) and zero if otherwise (no). Respondents who answered “yes” were asked to report the number of manufactured cigarettes smoked daily, which is known as the smoking intensity. This study uses these variables as dependent variables in investigating the socioeconomic determinants of tobacco smoking in the KSA.

Other socioeconomic and demographic characteristics, such as income, gender, age, marital status, education, and the region of residence, are used as independent variables, with income and education being used as the SES indicators among the respondents. Monthly income (Saudi Riyal, SR 1 = USD 0.27) is sorted into eight categories: ˂SR 3000 (reference); SR 3000 to less than 5000; SR 5000 to less than 7000; SR 7000 to less than 10,000; SR 10,000 to less than 15,000; SR 15,000 to less than 20,000; SR 20,000 to less than 30,000, and ≥SR 30,000.

Education is grouped as below primary school (reference), primary school, intermediate school, high school, and higher education. Age is measured as a continuous variable. Gender takes the value of one if the respondent is a male and zero for female. Marital status is also captured as a binary variable, whereby it takes the value of one for unmarried (including never-married, divorcees, separated and widowed) respondents and zero if married. To account for the regional differences in smoking patterns, we include regional variables in the regressions. This includes the 13 administrative regions in the KSA: Riyadh (reference), Madinah, AlBaha, Al-Jouf, Aseer, Eastern Region, Hail, Jezan, Najran, Northern Borders, Qaseem, Tabouk, and the Western Region.

### 2.3. Statistical Analysis

In order to investigate the socioeconomic factors influencing smoking, we estimate multivariate logistic regression (for the decision to smoke) and negative binomial regression (for smoking intensity) models, as these approaches are in line with the literature [16,27]. Additionally, the Wagstaff et al. [28] CIs are used to examine SES inequalities in smoking, whereby, using income, a positive CI indicates that smoking is disproportionately concentrated among the rich, while a negative CI indicates concentration among the poor. Similarly, when using education, a negative CI indicates that smoking is concentrated among the less educated. The education and income categories are used as ranking variables.

Prior to the multivariate analysis, a bivariate analysis using a Pearson Chi-square test of association is conducted. For ease of interpretation in percentages, we use (Odds Ratio−1)×100 for the logit model and $\;((e^{\mathrm{coefficient}})-1)\times 100$ for the negative binomial regression. As a robustness check, we use a two-part model to analyze the data. The results from the robustness checks are presented in the Appendix A.

## 3. Results

### 3.1. Descriptive Statistics

Of the total sample of 8813 respondents, 14.09% are current smokers (Table 1). Males represent 51.99% of the respondents and 32.63% are unmarried. For education, 18.67% of the respondents have no schooling or are below primary education, while 26.11% have completed higher education. On average, a smoker in our sample smokes 18.02 cigarettes per day, and the average age of the sample is about 39 years. Table 1 presents the other characteristics of the sample.

### 3.2. Bivariate Analysis

Prior to the multivariate estimations, as earlier indicated, a bivariate analysis of the association between current smoking status and the socioeconomic characteristics is conducted, and Table 2 shows the results of the bivariate analysis. The analysis shows that smoking is significantly associated with income (χ^2^ = 39.30, *p* < 0.01), while 38.37% and 8.88% of those who are currently smoking earn less than SR 7000 and SR 30,000 or more, respectively.

Table 2 also shows that there is a significant association between smoking and educational attainment (χ^2^ = 118.52, *p* < 0.01). Compared to highly educated people (14.65%) and those with a high school level of education (17.98%), the prevalence of current smoking is 6.20% among people with no schooling or who are below the primary school level of education. Moreover, smoking is significantly associated with gender (χ^2^ = 996.92, *p* < 0.01). Smoking is more concentrated among males (25.34%) than females (1.91%). Other factors that are significantly associated with smoking include marital status (χ^2^ = 12.57, *p* < 0.01) and region of residence (χ^2^ = 140.54, *p* < 0.01).

To determine the level of inequality in smoking in various socioeconomic groups (i.e., income and education), we estimate the Wagstaff et al. inequality CIs. The results are presented in Table 3.

At the national level, the income-based CI is 0.071 and that of education is 0.095. The CIs are statistically significant at the 1% level, which shows that overall smoking is concentrated among the socioeconomically better-off people in Saudi Arabia. Both the income- and education-based indices are qualitatively similar. By gender, income-based CIs are positive, but statistically insignificant. Among males, the education-based CI is 0.057 and is significant at the 5% level, while that of females is insignificantly negative.

There are regional variations in the disparities in smoking. For some regions, the inequality indices are positive. For instance, the income CIs in Hail (CI = 0.200, *p* < 0.01), Qassem (CI = 0.265, *p* < 0.01), Tabouk (CI = 0.121, *p* < 0.01) and the Western Region (CI = 0.122, *p* < 0.01) are positive and statistically significant. In Madinah and Aseer, the income CIs are negative and statistically insignificant at the conventional levels. The negative income CI in Riyadh is statistically significant at the 10% level.

**Figure 1:**
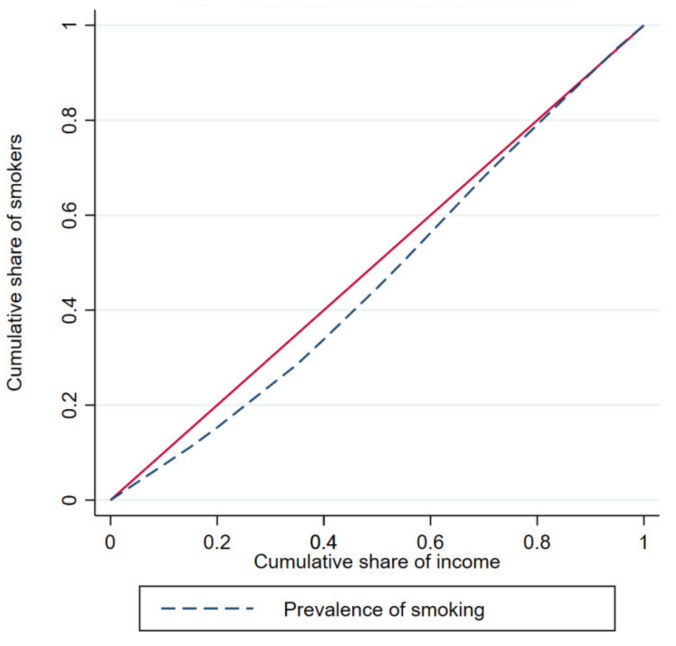
Income-based concentration curve.

**Figure 2:**
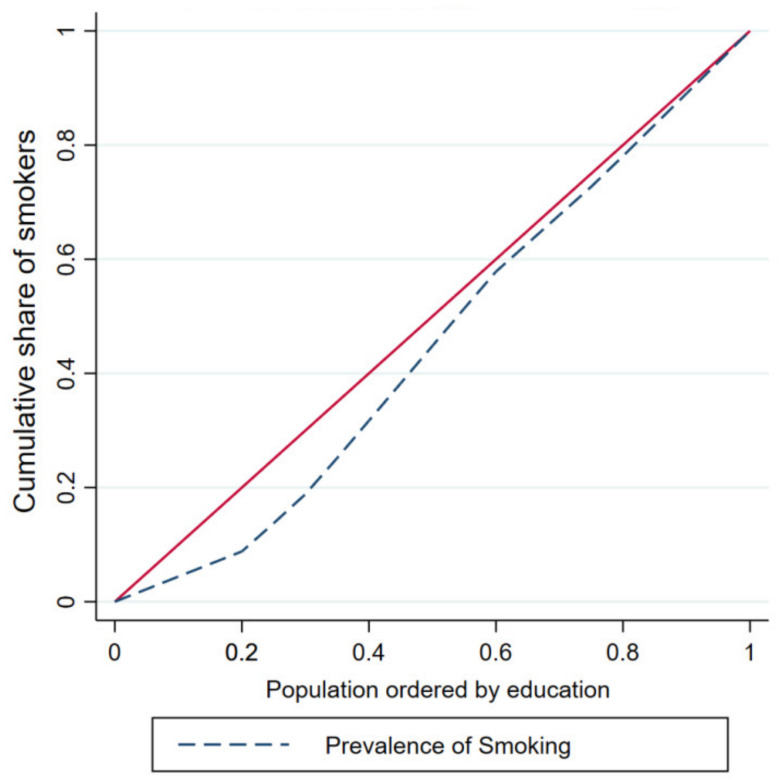
Education-based concentration curve.

Figure 1 and Figure 2 depict the concentration curves by income and education, respectively. The figures confirm that smoking is disproportionately concentrated among the wealthy and highly educated people in the KSA.

### 3.3. Regression Analysis

Given that univariate and bivariate analyses do not consider other variables that might influence the decision to smoke and the daily cigarette consumption (smoking intensity), this study adopts a multivariate analysis approach using logistic (logit) and negative binomial regression techniques. Table 4 presents the regression results on socioeconomic and demographic factors affecting the decision to smoke and smoking intensity in the KSA. Model 1 uses logit to estimate the decision to smoke, while Model 2 deals with the factors affecting smoking intensity using negative binomial regression. To account for selection bias, we use the two-part model to estimate the models simultaneously, and the results are presented in the Appendix A Table A1. The estimates from this model are qualitatively similar to those reported in Table 4.

The results from the logistic regression (Table 4, Model 1) show that, to some extent, SES measured by income is a significant determinant of the decision to smoke in the KSA. However, except for those earning SR 20,000 to less than SR 30,000, the odds ratio is below one for all income groups. For instance, the odds ratio is 0.447 and statistically significant at the 1% level when income is SR 30,000 or more. Moreover, respondents with incomes between SR 3000 and less than SR 5000, and between SR 10,000 and less than SR 15,000, have odds ratios of 0.809 and 0.779, respectively, and are both significant at the 10% level. In addition, the odds ratios decline as educational attainment increases, but are statistically insignificant. On the other hand, smoking intensity among smokers is not significantly influenced by the SES (income and education) of the respondents (Table 4, Model 2).

Factors such as age, marital status, gender and region of residence are found to significantly influence the decision to smoke (Table 4, Model 1). For instance, the odds ratio for age is 1.215 and is statistically significant at the 1% level, which implies that any additional year increases the likelihood of smoking by approximately 22%. This factor, however, peaks at a point and starts to decline, as shown by the quadratic age term (OR = 0.998, *p* < 0.01). Males are more likely to smoke (OR = 19.960, *p* < 0.01) than females.

In Model 2 (the smoking intensity model), the coefficient of age is 0.028, and is statistically significant at the 1% level. This means that any additional year is associated with a 2.8 higher smoking intensity. Compared to females, smoking intensity is 52.95% higher among males, as revealed by the coefficient in Model 2.

Marital status is another factor influencing the likelihood of smoking, with unmarried people having a higher likelihood of smoking (OR = 1.532, *p* < 0.01) compared to married people. Specifically, unmarried people are 53.2% more likely to smoke relative to their married counterparts. Nonetheless, we find no statistically significant difference in daily cigarette consumption rates between married and unmarried people (Table 4, Model 2). The odds ratios for the regional dummies suggest that there are regional differences in the likelihood of smoking and smoking intensity. Specifically, residents in Al-Jouf (OR = 1.989, *p* < 0.01), the Northern Borders (OR = 1.920, *p* < 0.01), Tabouk (OR = 1.592, *p* < 0.01) and the Western Region (OR = 1.587, *p* < 0.01) are found to be more likely to smoke than those in Riyadh, while residents of Aseer (OR = 0.463, *p* < 0.01) and Najran (OR = 0.398, *p* < 0.01) are found to be less likely to smoke relative to those in Riyadh. With regard to the intensity of smoking, we found that the daily number of cigarettes smoked is significantly lower in Aseer (β = −0.256, *p* < 0.01), the Eastern Region (β = −0.292, *p* < 0.01), Jezan (β = −0.238, *p* < 0.01) and Najran (β = −0.434, *p* < 0.01), relative to Riyadh.

## 4. Discussion

In this study, we examine the socioeconomic determinants and disparities in smoking in the KSA using the 2013 SHIS, which is a nationally representative survey. The results reveal that 14.09% of the respondents aged 15 years or older smoke cigarettes, and the average smoking intensity is 18.02 cigarettes per day. This high proportion of smokers in the KSA could be attributed to the absence of excise taxes during the period of the study. Overall, our hypothesis with regard to the existence of a statistically significant association between socioeconomic status and smoking (as well as its associated inequalities) is confirmed. Specifically, we find that smoking is concentrated among the socioeconomically better-off Saudis, which could be attributed to the lack of excise taxes, coupled with growing income, making tobacco products affordable. This finding is supported by another study in Namibia, where the prevalence and intensity of smoking were found to be pro-rich [21]. Conversely, the findings conflict with studies in sub-Saharan Africa [18], Argentina [19], and a sample of low-and middle-income countries [20], which found smoking to be more prevalent among those with lower socioeconomic status. There are also regional disparities in smoking, while the prevalence of smoking and the intensity are higher among Saudi men than women. Men have higher odds of smoking than women. They smoke 18.18 cigarettes per day, while women smoke 12.48 cigarettes daily. This may be because men are more likely to adopt risky behaviors relative to women. Our findings on gender differences in smoking are consistent with earlier studies in the KSA [2,3,4] and studies in Ghana and South Africa [16,27].

In addition, age and region of residence are found to influence the decision to smoke and the number of cigarettes smoked daily, while income and marital status are found to influence only the decision to smoke. The logistic regression estimates also imply that education is not a significant determinant of smoking and smoking intensity. Specifically, as regards age, the findings suggest that the likelihood of smoking increases as a person ages, but declines among the very old, and that the same can be said of smoking intensity. The reason for this is that many smokers start at a young age and, given that many smoking-related diseases manifest at a latter age [29], there is a lower likelihood that older people will take up smoking. In addition, current smokers might reduce their smoking intensity due to smoking-related health complications or intention to quit. Similar findings on the link between age and smoking have been reported in the literature [3,4,16].

Marital status is a significant determinant of the decision to smoke in the KSA. Unmarried people are more likely to smoke. However, there is no statistically significant difference between the smoking intensity for married and unmarried people. The finding that married people are less likely to smoke could be due to the KSA’s cultural setting, wherein married men are supposed to have control over their spouses and, hence, might restrict their female partners from smoking. Another reason is that some of the unmarried people in our sample are divorcees, separated or widowed, and such people might experience high levels of depression. This factor also partly explains the inconsistencies between our estimated odds and those in another study, which found that the likelihood of being a current smoker was higher among married, separated, divorced and widowed people, compared to the never-married people from the same SHIS data [2]. Surprisingly, in a recent study, no significant association was found between marital status and smoking [4].

In this study, the likelihood of smoking varies across the 13 administrative regions of Saudi Arabia, and it is consistent with the findings from a recent survey [4]. Compared to Riyadh, residents in Al-Jouf, the northern borders, Tabouk and the Western Region are more likely to smoke, while those in regions like Aseer and Najran are less likely to smoke. The daily number of cigarettes smoked is also significantly lower in Aseer, the Eastern Region, Najran and Jezan, relative to Riyadh. These differences may also reflect differences in the socioeconomic and demographic characteristics of the regions.

## 5. Conclusions

This study examines the socioeconomic and demographic determinants of smoking and smoking intensity in the KSA. Consistent with the literature, we find that smoking rates are significantly influenced by income, gender, age, marital status and region of residence. Men have higher odds of smoking than women and, if men decide to smoke, their intensity is 52.95% higher than that of women. We also find that unmarried people have higher odds of smoking. The inequality analysis shows that, overall, smoking is concentrated among the socioeconomically (income and education) better off.

In 2017, the Saudi government levied excise taxes on tobacco products, which was reviewed in 2019 [12]. In addition, smoking in public places and the sale of cigarettes to minors are prohibited to control smoking [2,6]. These policies are steps in the right direction. However, the implication of our findings calls for the government to institute targeted measures to curb tobacco use in Saudi Arabia. Thus, policies and measures aimed at effectively reducing tobacco consumption should pay attention to socio-demographic factors, such as gender, age, marital status and region of residence. Moreover, excise taxes aimed at making tobacco less affordable should also focus more on those with higher SES, since smoking prevalence is found to be more concentrated among them.

In spite of the above findings and recommendations, this study is not without limitations. The SHIS was a self-reporting survey and is therefore subject to recall bias. In addition, the SHIS was restricted to only Saudi Arabia; as such, the findings of the present study cannot be extended to other countries. In addition, there are other important factors affecting the decision to smoke, which the survey did not capture. For instance, exposure to tobacco adverts and anti-smoking campaigns, as well as changing community norms regarding smoking and taxation, are key factors that might influence smoking participation, but these are not included in the regressions because they were not captured in the survey. Therefore, future research should consider multi-country analyses as well as factors such as exposure to tobacco adverts and anti-smoking campaigns, among others if possible.

## Figures and Tables

**Table 1:** Descriptive statistics of the sample.

	Frequency (*n* = 8813)	%
**Smoking Prevalence**	1242	14.09
**Gender**		
Female	4231	48.01
Male	4582	51.99
**Marital Status**		
Unmarried	2876	32.63
Married	5937	67.37
**Education**		
None/Below Primary School	1645	18.67
Primary School	909	10.31
Intermediate School	1372	15.57
High School	2586	29.34
Higher Education	2301	26.11
**Monthly income level**		
<SR 3000	1529	17.35
SR 3000 to less than 5000	1620	18.38
SR 5000 to less than 7000	1444	16.38
SR 7000 to less than 10,000	1675	19.01
SR 10,000 to less than 15,000	1415	16.06
SR 15,000 to less than 20,000	660	7.49
SR 20,000 to less than 30,000	256	2.90
≥SR 30,000	214	2.43
**Region**		
Madinah	547	6.21
AlBaha	566	6.42
Al-Jouf	357	4.05
Aseer	767	8.70
Eastern Region	629	7.14
Hail	564	6.40
Jezan	725	8.23
Najran	635	7.21
Northern Borders	464	5.26
Qaseem	284	3.22
Riyadh	1408	15.98
Tabouk	479	5.44
Western Region	1388	15.75
Average smoking intensity (Both gender)	18.02 (Standard Deviation = 8.95)	
Average smoking intensity (Males)	18.18 (Standard Deviation = 8.94)	
Average smoking intensity (Females)	12.48 (Standard Deviation = 7.46)	
Average age	38.62 (Standard Deviation = 15.94)	

**Table 2:** Bivariate analysis of current smoking and categorical socioeconomic variables.

	*N*	Percent that Smoke	Chi-Square
**Gender**			996.92 ***
Female	4231	1.91	
Male	4582	25.34	
**Marital status**			12.57 ***
Unmarried	2876	12.20	
Married	5937	15.01	
**Education**			118.52 ***
None/Below Primary School	1645	6.20	
Primary School	909	14.96	
Intermediate School	1372	14.72	
High School	2586	17.98	
Higher Education	2301	14.65	
**Monthly income level**			39.30 ***
<SR 3000	1529	10.60	
SR 3000 to less than 5000	1620	12.47	
SR 5000 to less than 7000	1444	15.30	
SR 7000 to less than 10,000	1675	16.60	
SR 10,000 to less than 15,000	1415	15.27	
SR 15,000 to less than 20,000	660	14.85	
SR 20,000 to less than 30,000	256	17.97	
≥SR 30,000	214	8.88	
**Region**			140.54 ***
Madinah	547	14.08	
AlBaha	566	12.54	
Al-Jouf	357	20.73	
Aseer	767	6.00	
Eastern Region	629	16.85	
Hail	564	15.96	
Jezan	725	11.03	
Najran	635	6.14	
Northern Borders	464	20.47	
Qaseem	284	14.08	
Riyadh	1408	13.28	
Tabouk	479	18.37	
Western Region	1388	17.94	

*** *p* < 0.01.

**Table 3:** Wagstaff et al. inequality indices, by income and education, for smoking.

	Income		Education	
	CI Estimates	95% CI	CI Estimates	95% CI
**National Level**	0.071 ***	0.037 to 0.106	0.095 ***	0.061 to 0.128
**Gender**				
Female	0.009	−0.116 to 0.134	−0.042	−0.165 to 0.082
Male	0.023	−0.015 to 0.061	0.057 **	0.019 to 0.094
**Regions**				
Madinah	−0.035	−0.172 to 0.102	0.076	−0.06 to 0.211
AlBaha	0.016	−0.126 to 0.158	0.195 ***	0.056 to 0.334
Al-Jouf	0.013	−0.133 to 0.159	−0.044	−0.186 to 0.099
Aseer	−0.076	−0.246 to 0.094	0.131	−0.036 to 0.298
Eastern Region	−0.108 *	−0.227 to 0.010	−0.127 **	−0.243 to −0.011
Hail	0.200 ***	0.074 to 0.328	0.197 ***	0.071 to 0.323
Jezan	0.158 **	0.026 to 0.289	0.037	−0.094 to 0.168
Najran	0.232 **	0.050 to 0.414	0.413 ***	0.234 to 0.594
Northern Borders	0.163 **	0.035 to 0.291	−0.013	−0.139 to 0.114
Qaseem	0.265 ***	0.076 to 0.454	0.246 **	0.059 to 0.433
Riyadh	−0.073 *	−0.161 to 0.014	−0.020	−0.106 to 0.065
Tabouk	0.121 ***	−0.009 to 0.252	0.256 ***	0.128 to 0.385
Western Region	0.122 ***	0.044 to 0.200	0.06	−0.017 to 0.136

*** *p* < 0.01, ** *p* < 0.05, * *p* < 0.1.

**Table 4:** Socioeconomic determinants of smoking and smoking intensity.

Variables.	Model 1	Model 2
	Decision to Smoke	Smoking Intensity
	Logit	Negative Binomial
	Odds Ratio	Coefficients
**Gender**		
Female	Ref	Ref
Male	19.960 ***	0.425 ***
	(2.422)	(0.108)
**Marital Status**		
Married	Ref	Ref
Unmarried	1.532 ***	0.073
	(0.152)	(0.046)
**Age**	1.215 ***	0.028 ***
	(0.019)	(0.008)
**Age squared**	0.998 ***	−0.000 ***
	(0.000)	(0.000)
**Education**		
None/Below Primary School	Ref	Ref
Primary School	1.164	−0.052
	(0.183)	(0.078)
Intermediate School	1.067	−0.054
	(0.159)	(0.073)
High School	1.052	−0.048
	(0.163)	(0.075)
Higher Education	0.907	−0.069
	(0.143)	(0.078)
**Monthly income level**		
<SR 3000	Ref	Ref
SR 3000 to less than 5000	0.809 *	−0.034
	(0.102)	(0.060)
SR 5000 to less than 7000	0.909	−0.050
	(0.117)	(0.061)
SR 7000 to less than 10,000	0.928	−0.037
	(0.118)	(0.060)
SR 10,000 to less than 15,000	0.779 *	−0.051
	(0.106)	(0.064)
SR 15,000 to less than 20,000	0.787	−0.112
	(0.129)	(0.080)
SR 20,000 to less than 30,000	1.008	−0.040
	(0.213)	(0.108)
≥SR 30,000	0.447 ***	0.162
	(0.124)	(0.131)
**Region**		
Riyadh	Ref	Ref
Madinah	0.904	0.029
	(0.142)	(0.075)
AlBaha	1.230	0.047
	(0.200)	(0.076)
Al-Jouf	1.989 ***	0.029
	(0.340)	(0.086)
Aseer	0.463 ***	−0.256 ***
	(0.084)	(0.093)
Eastern Region	1.255	−0.292 ***
	(0.181)	(0.074)
Hail	1.253	0.080
	(0.191)	(0.071)
Jezan	1.145	−0.238 ***
	(0.180)	(0.086)
Najran	0.398 ***	−0.434 ***
	(0.077)	(0.100)
Northern Borders	1.920 ***	−0.136*
	(0.299)	(0.069)
Qaseem	1.262	−0.130
	(0.261)	(0.101)
Tabouk	1.592 ***	0.181 **
	(0.250)	(0.072)
Western Region	1.587 ***	−0.045
	(0.184)	(0.059)
Constant	0.000 ***	1.892 ***
	(0.000)	(0.217)
Observations	8,813	900
Pseudo R-squared	0.218	0.018
Chi2	1563 ***	117.9 ***

Standard errors in parentheses; *** *p* < 0.01, ** *p* < 0.05, * *p* < 0.1.

## Data Availability

The datasets generated and/or analyzed during the current study are not publicly available due to privacy, confidentiality, and other restrictions. Access to data can be gained through the Ministry of Health in Saudi Arabia.

## Appendix A

**Table A1:** Socioeconomic determinants of smoking and smoking intensity using a two-part model.

Variables	Decision to Smoke	Smoking Intensity
	Logit	Negative Binomial (GLM)
	Odds Ratio	Coefficients
**Gender**		
Female	Ref	Ref
Male	45.439 ***	0.425 *
	(9.427)	(0.221)
**Marital Status**		
Married	Ref	Ref
Unmarried	1.610 ***	0.075
	(0.182)	(0.098)
**Age**	1.209 ***	0.029 *
	(0.022)	(0.016)
**Age squared**	0.998 ***	−0.000
	(0.000)	(0.000)
**Education**		
None/Below Primary School	Ref	Ref
Primary School	1.119	−0.058
	(0.201)	(0.166)
Intermediate School	1.003	−0.055
	(0.172)	(0.156)
High School	1.045	−0.050
	(0.184)	(0.161)
Higher Education	0.773	−0.071
	(0.141)	(0.166)
**Monthly income level**		
<SR 3000	Ref	Ref
SR 3000 to less than 5000	0.709 **	−0.038
	(0.099)	(0.127)
SR 5000 to less than 7000	0.786 *	−0.054
	(0.112)	(0.128)
SR 7000 to less than 10,000	0.800	−0.038
	(0.113)	(0.126)
SR 10,000 to less than 15,000	0.691 **	−0.056
	(0.105)	(0.134)
SR 15,000 to less than 20,000	0.662 **	−0.119
	(0.123)	(0.169)
SR 20,000 to less than 30,000	0.729	−0.047
	(0.181)	(0.230)
≥SR 30,000	0.512 **	0.160
	(0.152)	(0.282)
**Region**		
Riyadh	Ref	Ref
Madinah	0.940	0.024
	(0.162)	(0.161)
AlBaha	1.355 *	0.047
	(0.241)	(0.162)
Al-Jouf	1.330	0.030
	(0.266)	(0.182)
Aseer	0.474 ***	−0.260
	(0.096)	(0.195)
Eastern Region	0.960	−0.297 *
	(0.160)	(0.156)
Hail	1.238	0.078
	(0.208)	(0.152)
Jezan	0.761	−0.242
	(0.145)	(0.179)
Najran	0.440 ***	−0.437 **
	(0.093)	(0.205)
Northern Borders	2.108 ***	−0.139
	(0.354)	(0.147)
Qaseem	1.153	−0.133
	(0.269)	(0.212)
Tabouk	1.603 ***	0.180
	(0.276)	(0.153)
Western Region	1.233	−0.047
	(0.163)	(0.124)
Constant	0.000 ***	1.882 ***
	(0.000)	(0.456)
Observations	8813	900
Pseudo R-squared	0.224	
Chi2	1303 ***	

Standard errors in parentheses; *** *p* < 0.01, ** *p* < 0.05, * *p* < 0.1.
